# Morphometric analysis of the splenic artery using contrast-enhanced computed tomography (CT)

**DOI:** 10.1007/s00276-020-02598-1

**Published:** 2020-10-26

**Authors:** David J. Brinkman, Stephanie Troquay, Wouter J. de Jonge, Eric D. Irwin, Margriet J. Vervoordeldonk, Misha D. P. Luyer, Joost Nederend

**Affiliations:** 1 Department of Surgery, Catharina Hospital, Postbus 1350, 5602 ZA Eindhoven, The Netherlands; 2 Tytgat Institute for Liver and Intestinal Research, Amsterdam UMCUniversity of Amsterdam, Amsterdam, The Netherlands; 3 Department of Radiology, Catharina Hospital, Eindhoven, The Netherlands; 4 Galvani Bioelectronics, Stevenage, UK; 5 Department of Surgery, University of Minnesota Medical School, Minneapolis, USA

**Keywords:** Splenic artery, Tortuosity, Computed tomography, Neuromodulation

## Abstract

**Purpose:**

To evaluate the morphology and course of the splenic artery, which might impact the surgical implantation of systems that stimulate the nerves surrounding the splenic artery. Experimental studies indicate that these nerves play an important part in immune modulation, and might be a potential target in the treatment of autoimmune diseases.

**Methods:**

This retrospective cohort study made use of contrast-enhanced CT images from 40 male and 40 female patients (age 30–69) that underwent a CT examination of the aorta, kidneys or pancreas. Anatomic features were described including total splenic artery length, calibers, tortuosity, the presence of arterial loops and the branching pattern of the splenic artery.

**Results:**

No age-gender-related differences could be found related to tortuosity or branching pattern. The length of splenic artery in contact with pancreatic tissue decreased with increasing age, but was not different between genders. Artery diameters were wider in male compared to female subjects. Loops of variable directions, that represent a part of the artery that curls out of the pancreatic tissue, were identified in each age-gender category and were present in nearly all subjects (86%).

**Conclusion:**

This study suggests that although some anatomic features of the splenic artery are subject to factors as age and gender, the tortuosity of the splenic artery is not age dependent. Most subjects had one or multiple loops, which can serve as a target for neuromodulatory devices. Future studies should investigate whether splenic nerve stimulation is safe and feasible.

## Introduction

There is a growing body of experimental data to support the hypothesis that electrical stimulation of the nerves that project to the spleen can modulate immune responses, making it a potential target for neuromodulation in the treatment of chronic inflammatory conditions [5, 14, 17]. These nerves form a plexus around the splenic artery before innervating the spleen [3, 6]. Therefore, the course of the splenic artery can impact the therapeutic potential of splenic nerve stimulation in humans.

The study of splenic artery anatomy has been complicated by the potential effect of aging on splenic artery tortuosity, as well as the effect of using different methodologies for characterizing the arterial anatomy [15]. An example of this is the assessment of proximity of the splenic artery to the pancreas where gross anatomic and radiographic studies have demonstrated different results [4, 11, 19]. In an autopsy series, Pandey et al. reported that the splenic artery was within the substance of the pancreas in 23.1% cases while in a cohort of patients studied by cross-sectional imaging Zhu and colleagues reported this relationship in 63.3% of cases. Determining the actual area where the splenic artery is in direct contact with the pancreas could provide a better characterization of their anatomic relationship and might help to indicate if placement of a neuromodulatory device is feasible.

It is thought that the splenic artery elongates and becomes more tortuous with age, which increases separation between the splenic artery and the pancreas [15]. This increased separation may allow a safer approach for device implantation on the splenic artery, lymphadenectomy, or to select cases for vessel sparing distal pancreatectomy. Our understanding of this important anatomic detail is; however, largely derived from cadaveric studies, where degradation and formalin fixation may result in altered vascular and pancreatic anatomy [4, 15, 18]. Furthermore, it is not systematically described in literature how splenic artery diameters vary between splenic origin and branching the splenic hilum, which can impact design of neuromodulatory devices.

To address these limitations, and for the preparation of a clinical trial (www.clinicaltrials.gov; NCT04171011), we have undertaken a study to characterize arterial tortuosity, branching, and anatomic proximity of the artery to the pancreas in a single cohort of patients using 3D reconstructed images from abdominal CT angiograms. Furthermore, we addressed the effects of age and gender difference on these anatomic features.

## Materials and methods

### Subjects

The data for this study were collected using randomly chosen contrast-enhanced CT images from subjects that underwent a CT examination of the aorta, kidneys, or pancreas in the Catharina Hospital Eindhoven, the Netherlands between January 1, 2009 and January 1, 2018. All CT scans were acquired with an iCT scanner (Koninklijke Philips N.V., the Netherlands). Images were obtained using a standardized protocol. Scanning conditions were a voltage of 100 kV, gantry rotation time of 0.75 s, pitch of 0.914, and automated detector collimation. Slice thickness was 0.7 mm. Field of view (FOV) was 250–500 mm dependent on patient size with a 512 matrix size. Multiplanar reconstructions were available. Subjects received between 80 and 120 mL nonionic contrast medium (Iomeron 300, Bracco, Italy). The Medical Research Ethics Committee United (MEC-U, Nieuwegein, The Netherlands) approved the study (reference number W18.127) and waived the requirement of informed consent. In compliance with the EU General Data Protection Regulation (GDPR), subjects received a letter of notification that their data was used in this study. If there were any objections, subjects could refuse the use of their data. CT images were selected by an experienced radiologist (JN) and assessed for quality (adequate opacification of the splenic artery, and the absence of artifacts that would impact the reading and interpretation of the CT images). Exclusion criteria were previous pancreatic or splenic surgery, pancreatic cancer, splenomegaly, spleen cancer, and Hodgkin lymphoma. In addition, patients with conditions that affect normal vascular anatomy or interfered with the performing the necessary measurements, were excluded from the study at the discretion of the analysts or supervising radiologist. Subjects (sex ratio 1:1) were proportionately allocated in four age decades between 30 and 69 years (30–39, 40–49, 50–59 and 60–69). Because of the limited availability of CT imaging in younger patients, a lower limit of 30 years was chosen. An upper limit of 69 years was selected to create a study population representing the target group for potential neuromodulatory device implantation.

### Data collection

Scans from selected subjects were analyzed using image analysis software (Intellispace Portal, Koninklijke Philips N.V., The Netherlands). Image analysis was performed by two qualified registrars (DB and ST), supervised by the radiologist (JN) for quality assurance. The data were systemically acquired using electronic case report forms (Research Manager, de research manager, Deventer, The Netherlands). Subject characteristics, if available, were extracted from the clinical record, including age, height, weight, and body mass index.

### Measurements

**Fig. 1:**
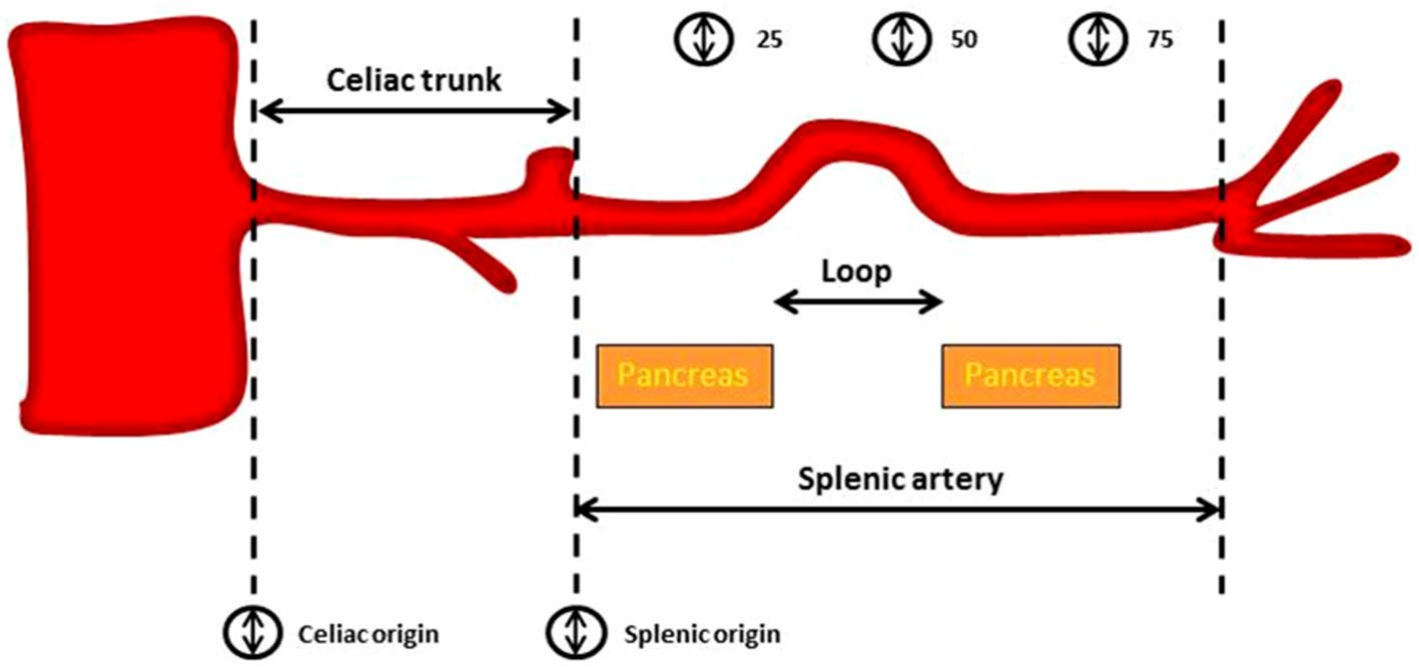
Schematic overview of the measurements of the celiac trunk and splenic artery   = caliber

It was determined whether each splenic artery originated from the celiac trunk. Maximum calibers were measured at the origins of the celiac and splenic arteries and at predefined points related to the origin of the splenic artery (25, 50 and 75% of the total splenic artery length). The total length of the splenic artery was defined as the length through the center of the vessel from the origin of the splenic artery to the point in the splenic hilum where main branching occurred. The relation of the splenic artery to the pancreas was described as the part of the splenic artery that is in direct contact with the pancreas as a percentage of the total splenic artery length, i.e., no distinct layer of tissue was present between the artery and pancreas (Fig. 1). The tortuosity index of the splenic artery was calculated by the analysis software, normalizing the uncoiled length of the artery between the origin of the splenic artery and its termination in the splenic hilum, defined as *Y*, to the linear distance between the origin of the splenic artery and its termination in the splenic hilum, defined as *X*. The tortuosity index is calculated as the ratio (*Y*/*X*) [15]. The branching pattern in the splenic hilum was described. Furthermore, incidence of proximal polar branching was determined, i.e., arterial branches that branch off to the splenic poles before main branching in the splenic hilum. Splenic artery loops were defined as segments of the splenic artery, where there was a distinct layer of tissue, between the artery and the pancreas and because of its direction, easy to access during laparoscopy. To give a better idea on where each loop is located, a distinction was drawn between proximal and distal loops. Proximal loops are approximately located in the first 50% of the splenic artery and distal loops in the last 50%.

### Statistical analysis

Prism 8.3 (GraphPad Software, La Jolla, CA) was used to create graphs and perform statistics. The data were tested for normality, but since the majority of data was non-normally distributed, all the data were presented as median [interquartile range], and compared with the Mann–Whitney *U* test if needed. In case of multiple groups, a Kruskall–Wallis and Dunn’s multiple comparison test were used. Association between tortuosity index and other variables was tested by means of Spearman’s rank correlation coefficient. Female and male patients were combined in four age categories to assess the influence of age on the presence of loops (0 or ≥ 1) with the Fisher’s exact test. A *P* value < 0.05 was considered statistically significant.

## Results

### Study population and splenic artery characteristics

**Fig. 2:**
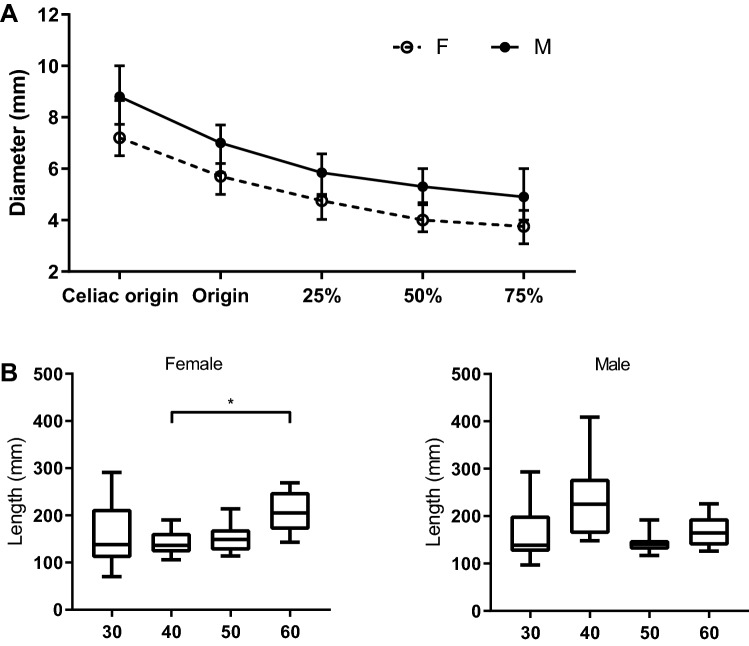
Splenic artery caliber at standardized locations. **a** The data are presented as median with interquartile range for both female and male subjects. **b** The data are presented as median (line), interquartile range (boxes), and range (whiskers). Statistically significant differences (*P* < 0.05) between age groups as showed by Dunn’s multiple comparison test are indicated by *

A total of 80 subjects were included. Subject characteristics, such as height, weight, and BMI are presented in Table 1. The total splenic artery length and the calibers of the splenic artery are depicted in Table 1 and Fig. 2. Each subject had a splenic artery that originated from the celiac trunk. The splenic artery tended to elongate with age in female subjects, with longer splenic artery lengths in the 60 group as compared to the 40 group (*P* = 0.02), while this trend was not seen in male subjects. Splenic artery diameter was greater in male subjects as compared to in female subjects at all predefined locations (*P* < 0.05). In both males and females, the caliber of the splenic artery is at its largest at the origin and becomes narrower during its course before branching in the splenic hilum.

**Table 1:** Subject and splenic artery characteristics for each age-gender category

	Female				Male			
	30–39 (30F)	40–49 (40F)	50–59 (50F)	60–69 (60F)	30–39 (30M)	40–49 (40M)	50–59 (50M)	60–69 (60M)
Length (cm)	169 [157–176]	170 [165–172]	171 [169–172]	165 [162–168]	183 [176–186]	182 [181–187]	178 [171–183]	178 [176–185]
Weight (kg)	61 [53–67]	72 [63–85]	65 [59–77]	79 [71–93]	81 [67–91]	93 [88–99]	94 [83–109]	91 [83–109]
BMI (kg/m^2^)	21.1 [20.2–22.9]	24.3 [21.3–29.9]	23.0 [20.0–25.2]	29.2 [24.8–34.6]	25.1 [20.2–26.3]	28.0 [26.4–30.7]	28.7 [27.7–33.4]	27.6 [24.5–32.7]
Splenic artery length (cm)	138 [109–214]	137 [122–163]	149 [125–171]	205 [170–250]	139 [124–202]	225 [162–279]	141 [129–150]	165 [139–196]
Celiac origin (mm)	6.9 [6.1–8.1]	8.0 [7.1–8.9]	7.0 [6.0–8.0]	8.0 [6.7–9.4]	8.8 [7.7–9.4]	8.4 [7.4–10.1]	8.5 [7.0–9.3]	9.9 [8.0–10.3]
Splenic origin (mm)	5.9 [4.9–6.5]	5.5 [4.9–5.9]	5.5 [5.0–6.3]	5.3 [4.8–6.9]	6.7 [6.3–7.8]	7.2 [5.6–7.7]	7.0 [5.8–7.3]	6.4 [5.4–7.1]
Caliber at 25% of length (mm)	4.9 [3.9–5.0]	4.2 [3.4–4.6]	5.0 [4.0–5.0]	4.7 [3.9–5.9]	5.7 [5.1–6.4]	5.1 [4.0–7.1]	6.0 [5.8–7.3]	5.3 [4.5–6.3]
Caliber at 50% of length (mm)	4.1 [3.5–4.7]	4.0 [3.5–4.3]	4.5 [4.0–5.3]	4.0 [3.0–4.9]	5.3 [4.4–5.6]	5.3 [4.6–6.2]	6.0 [5.0–6.0]	4.6 [4.1–6.5]
Caliber at 75% of length (mm)	4.2 [3.6–4.7]	3.5 [2.9–3.7]	4.0 [3.0–5.0]	3.6 [3.6–4.6]	4.2 [3.8–6.0]	4.7 [4.1–5.7]	5.5 [4.0–6.3]	4.9 [4.0–6.0]
Loops								
No loops	4	1	0	0	4	1	1	0
1 loop	3	9	4	2	1	4	7	7
2 or more loops	3	0	6	8	5	3	2	3

The data are presented as median [IQR] or as count

### Relation to the pancreas and other organs

**Fig. 3:**
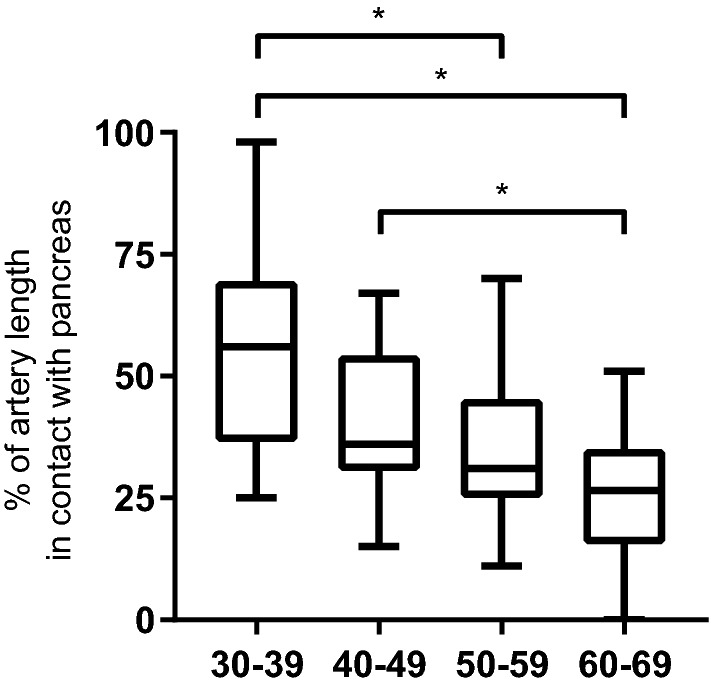
Percentage of total splenic artery length that is in contact with the pancreas. The data are presented as median (line), interquartile range (boxes), and range (whiskers). Kruskall–Wallis test showed *P* < 0.0001. Statistically significant differences (*P* < 0.05) between age groups as showed by Dunn’s multiple comparison test are indicated by *

The percentage of total splenic artery length that is in contact with pancreatic tissue decreased with age (Fig. 3). In the study population, the splenic artery was rarely in contact with organs other than the pancreas. In 20% of subjects, a short segment of the splenic artery was in contact with the stomach and in one subject (1.2%) there was contact with the left kidney.

### Tortuosity

**Fig. 4:**
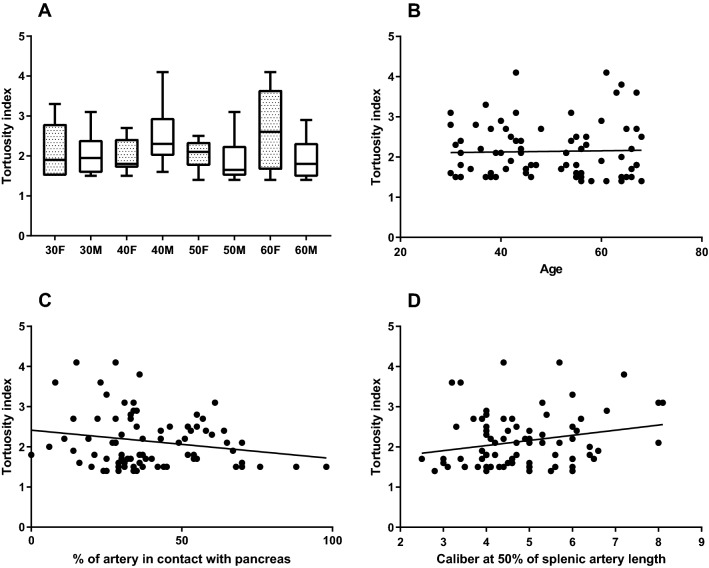
Tortuosity index of the splenic artery. Age-gender categories (**a**) and correlation of tortuosity index with age (**b**), contact with the pancreas (**c**) and calibers at 50% of total splenic artery length. The data are presented as median (line), interquartile range (boxes), and range (whiskers) (**a**) or plotted as one point for each subject (**b**–**d**)

The tortuosity index of the splenic artery is presented in Fig. 4 for all age-gender categories. There was a high variability within each age-gender category, and no correlation was observed between age and tortuosity index. Furthermore, the tortuosity index was not correlated with the percentage of the splenic artery that is in contact with the pancreas (*r* = − 0.12; *P* = 0.31) and although statistically significant, poorly correlated with the caliber of the splenic artery at 50% of the total length (*r* = 0.19; *P* = 0.04).

### Loops

**Fig. 5:**
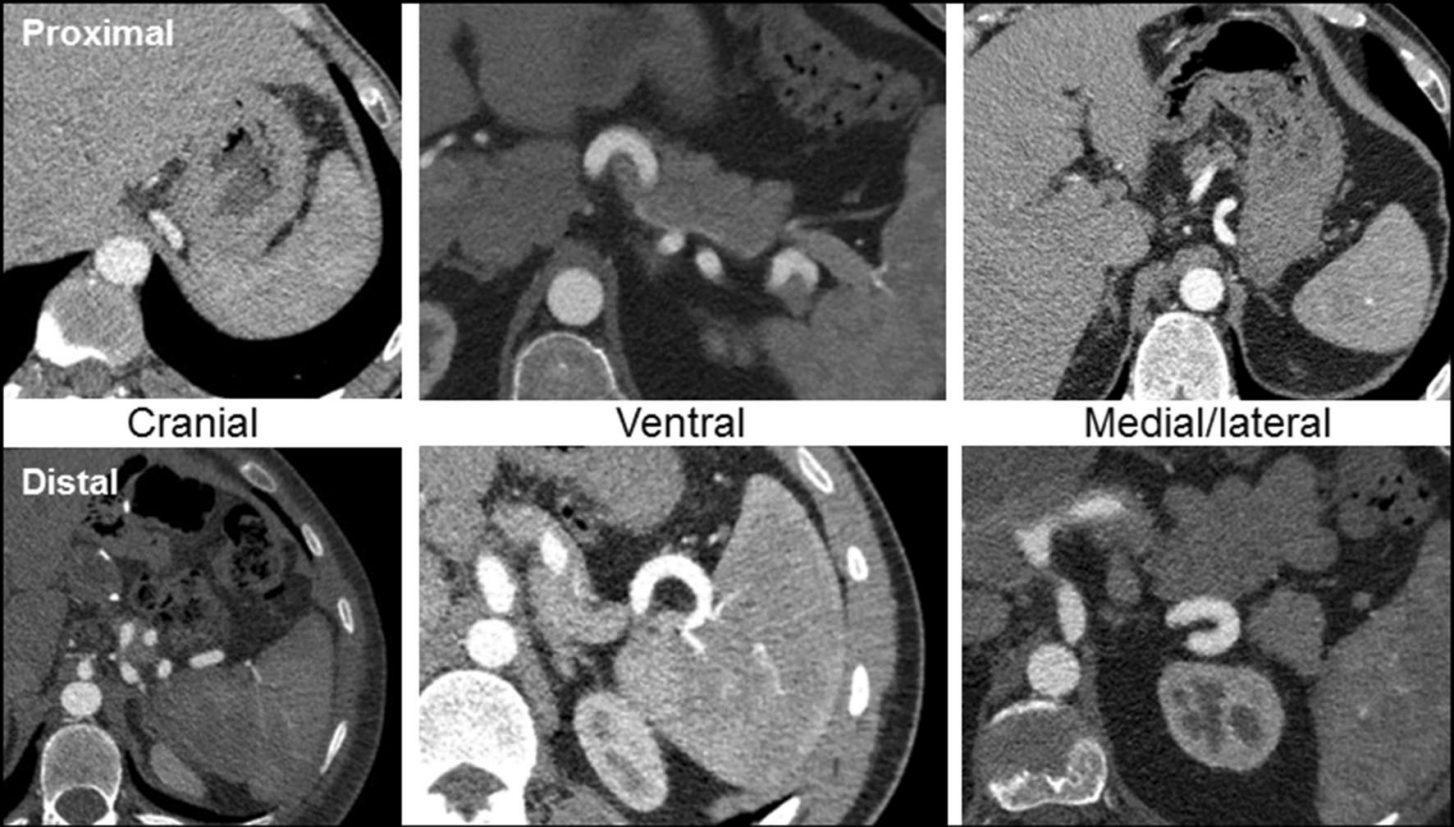
Common anatomic directions of proximal and distal splenic artery loops

The number of loops in each age-gender category is shown in Table 1. The portion of subjects without any loops decreased with age (*P* = 0.002). Arterial loops were evenly distributed between the proximal and distal splenic artery and the median diameters were comparable between proximal and distal loops (5.1 [4.5–6.3] vs. 5.0 [4.2–6.0] mm, respectively, *P* = 0.33). The anatomic paths taken by the loops were highly variable, see representative cross-sectional images (Fig. 5), although there more loops oriented cranially as the arteries traveled away from the pancreas.

### Branching pattern

For the total study population, the median number of branches of the main splenic artery, in the splenic hilum, was 2 [2, 3]. In 59% of subjects (47/80), there were also branches from the proximal splenic artery that coursed to the hilum, projecting to the cranial or caudal pole of the spleen separate from the main splenic artery. The median length of these early branches to the splenic hilum was 37 mm (range 6–266 mm).

## Discussion

In this study, we used cross-sectional imaging to evaluate splenic artery morphology and relationships of the splenic artery to adjacent structures in a cohort of patients stratified by age and gender. We found that splenic artery loops as potential sites for neuromodulatory device placement were present in a large number of patients. We also identified a high degree of variability of splenic artery length, caliber, and tortuosity. We demonstrated a novel finding that the length of the splenic artery in direct contact with the pancreas decreases with advancing age. Remarkably, this observation was not the result of increasing arterial tortuosity which did not vary significantly between the different age-gender categories.

In this cohort of patients, the splenic artery was found to gradually decrease in diameter as it traveled from its origin until it terminated with branching in the splenic hilum. In addition, male subjects were found to have vessels with larger diameters than females. Although these observations have not been previously reported for the splenic artery, they are consistent with findings in the coronary bed, as well the upper extremity arteries [7, 12]. Our findings of splenic artery length and the pattern of terminal branching were consistent with previous studies [1, 2, 4, 18]. Another noteworthy observation in this study is the finding of proximal polar branches, in which an arterial branch arises directly from the main splenic artery, traveling to the cranial or caudal pole of the spleen without passing through the hilum. These were present in 59% of all subjects, which is remarkably higher than a different imaging study (5%), but in agreement with cadaver work (51%) [4, 18]. While these differences may reflect different protocols used for imaging, it is possible that these represent true differences between the patient populations rather than being accounted for by gender- or an age-related phenomenon.

The observations in this study not only add to our understanding of splenic artery morphology but also demonstrate the value of using CT angiography for defining splenic artery anatomy and its relationship to regional anatomic structures, which aids in treatment planning for a range of procedures such as distal pancreatectomy, lymphadenectomy for oncologic resections and endovascular interventions [9, 13, 18, 19]. Beyond these traditional indications, these observations may be important in the development of a new therapy, currently being investigated, which uses stimulation of the splenic plexus to modulate immune responses in subjects suffering from chronic inflammatory diseases [5]. In humans, the splenic neural plexus has been shown to run in close proximity with the splenic artery [16]. Furthermore, it has been shown that loops are surrounded by a substantial amount of nerve tissue [3]. The present study, using cross sectional imaging of living subjects, is the first to report on the anatomy of the splenic artery as it relates to contact with the pancreas and to explore this anatomy as it may impact accessibility of the splenic artery and associated nerve plexus. This is significant as this relationship impacts the ease of surgical dissection in the perivascular space while minimizing the potential for injury to local structures.

To accomplish this, the relationship between the splenic artery and pancreas were characterized by measuring the proportion of the total length of the splenic artery that is in contact with the pancreas as well as the presence and characteristics of splenic artery loops, segments where the artery is distinct from the pancreas. We demonstrated that the amount of the splenic artery that was in contact with the pancreas decreases with age. These observations did not correlate with increases in tortuosity or diameter, which is consistent with the hypothesis that this is not solely due to generalized arterial enlargement. Splenic artery loops, as described in the study, were present in most patients and equally distributed throughout the course of the splenic artery. This comparison may, however, have been limited by a large amount of variability within the age-gender categories. Comparison of these findings with those of other studies is difficult, since the relation to the pancreas was described in a different way. Zhu et al. used the most inferior part of the splenic artery as a reference point, whereas Zheng and colleagues described whether the splenic artery was located supra- or intra-/retropancreatically [18, 19]. It can however be stated that there is a high variation in these studies, since different rates of supra- and intrapancreatic courses are reported [11, 18, 19]. In our opinion, determining the actual area where the splenic artery is in direct contact with the pancreas provides a better characterization of the relationship between the splenic artery and the pancreas.

In contrast to what was found in cadaver studies, this study has been unable to demonstrate that the tortuosity of the splenic artery increases with age [4, 15]. This difference could be because this study was limited to patients who were between 30- and 69-years of age, while the cadaver studies also included fetuses, neonates and young children. Our study did not include data from children or young adults because of limited availability of clinical CT imaging in these age categories. As such, it cannot account for any increases in arterial dimensions or tortuosity that has been described in these younger patient groups [15]. Given the high variation in each age-gender category in this study, it seems likely that factors other than age have a greater influence on the development of tortuosity of the splenic artery. Alternatively, this may reflect difference between measurements taken in decompressed arteries as compared to those taken in vivo where they are distended by arterial pressure.

It is important to bear in mind the possible limitations to this study. First, this is a retrospective study of CT images previously acquired as part of the patient’s standard care. Underlying conditions of subjects could have had an influence on the course of the splenic artery, although an effort was made to identify factors with known relationships to altered anatomy and structure and to include these prospectively in the study’s exclusion criteria. Another limitation of this study design is that, by using angiography, only the caliber of the splenic artery lumen was measured, rather than the external diameter of the splenic artery including the arterial wall. This also limits the assessment of smaller arteries such as pancreatic branches. Histopathological studies and ultrasonography could provide additional information on the wall thickness of the splenic artery should total artery diameter be required [8, 10].

In summary, this study describes the characteristics of the splenic artery, with specific attention for age-gender differences and the relation to the pancreas. This study supports the idea that there are segments of the splenic artery that are discrete from the pancreas and are approachable at locations without causing significant damage to the pancreas or other structures. Clinical studies are ongoing to confirm that the splenic artery and nerve plexus are accessible and if a device can safely be implanted in a clinical surgical setting (www.clinicaltrials.gov; NCT04171011).
